# Comprehensive investigation of material properties and operational parameters for enhancing performance and stability of FASnI_3_-based perovskite solar cells

**DOI:** 10.1038/s41598-024-67418-7

**Published:** 2024-07-17

**Authors:** Rania Saleh Alqurashi

**Affiliations:** Department of Physics, Faculty of Science, Al-Baha University, 65779-7738 Alaqiq, Saudi Arabia

**Keywords:** Lead free perovskite, Performance, FASnI_3_, Optical properties, SCAPS-1D, Solar energy, Solar cells

## Abstract

Recent advancements in the efficiency of lead-based halide perovskite solar cells (PSCs), exceeding 25%, have raised concerns about their toxicity and suitability for mass commercialization. As a result, tin-based PSCs have emerged as attractive alternatives. Among diverse types of tin-based PSCs, organic–inorganic metal halide materials, particularly FASnI_3_ stands out for high efficiency, remarkable stability, low-cost, and straightforward solution-based fabrication process. In this work, we modelled the performance of FASnI_3_ PSCs with four different hole transporting materials (Spiro-OMeTAD, Cu_2_O, CuI, and CuSCN) using SCAPS-1D program. Compared to the initial structure of Ag/Spiro-OMeTAD/FASnI_3_/TiO_2_/FTO, analysis on current–voltage and quantum efficiency characteristics identified Cu_2_O as an ideal hole transport material. Optimizing device output involved exploring the thickness of the FASnI_3_ layer, defect density states, light reflection/transmission at the back and front metal contacts, effects of metal work function, and operational temperature. Maximum performance and high stability have been achieved, where an open-circuit voltage of 1.16 V, and a high short-circuit current density of 31.70 mA/cm^2^ were obtained. Further study on charge carriers capture cross-section demonstrated a PCE of 32.47% and FF of 88.53% at a selected capture cross-section of electrons and holes of 10^22^ cm^2^. This work aims to guide researchers for building and manufacturing perovskite solar cells that are more stable with moderate thickness, more effective, and economically feasible.

## Introduction

One of the ways to address the increasing global energy demands resulting from extensive infrastructure development is through investment in solar cell technology, where sunlight is converted into electricity using a photovoltaic cell. As a result, the production of solar cells experiences an average biannual increase. The structure of solar cells typically comprises a perovskite layer, where the absorption of light generates most charge carriers within the device. After the absorber layer, transport layers for holes and electrons facilitate the movement of formerly produced charge carriers directed at the anode and cathode^1^. These layers play a crucial role as they significantly contribute to the performance of solar devices.

Organic–inorganic metal halide perovskite solar cells (PSCs) have received significant attention among various solar cell technologies since firstly reported in 2009 by Miyasaka^2^. Their power conversion efficiency has rapidly increased from 3 to 24%, approaching the efficiency of silicon technology, which stands at around 27%^3,4^. Thanks to the unique structure of perovskite materials employing the ABX_3_ formula, where A represents the larger cation of either inorganic (alkali metal Cs^+^) or organic (CH_3_NH_3_^+^, MA or HC(NH_2_)_2_^+^, FA) nature, B is the smaller divalent metal cation (${\text{Pb}}^{2+}$, ${\text{Sn}}^{2+}$, or ${\text{Ge}}^{2+}$) and X denotes a single halide anion or mixture (${\text{F}}^{-}$, ${\text{Cl}}^{-}$, ${\text{Br}}^{-}$, or ${\text{I}}^{-}$)^2^. Careful choices of these cations and anions can improve device efficiency and stability. Bhattarai et al. reported using SCAPS-1D the optimized performance of devices with different cation and anion in the perovskite structure^5–9^.

The photoactive characteristics of perovskites demonstrate outstanding carrier mobilities ranging from 10 to 200 cm^2^V⁻^2^ s⁻^1^, minimal dipolar binding energies at 0.30 eV, extended carrier (holes and electrons) diffusion lengths exceeding 1 µm, impressive carrier lifetimes of 270 ns, remarkable tolerance to defect densities (up to 10^1^⁶ cm⁻^3^), a strong dielectric constant and bipolar transport characteristics^10^. Coupled with its adjustable band gap within the range of 1.24–1.7 eV, straightforward application through simple solution processes, cost-effectiveness, and ready availability, perovskite technology is rapidly advancing in photovoltaics^11^.

Nevertheless, there are still pertinent challenges that require attention. Various factors influence the performance of the material, encompassing considerations such as the toxicity of specific components in the device structure, the material’s phase stability during transitions, long-term stability, the trade-off between thickness and performance, the degree of crystallinity achieved through bulk reduction, management of interface defect density states and boundaries at the grains, resilience to exterior temperature fluctuations during operation, optimization of light collection through surface and interface engineering, and the effective collection and transportation of photogenerated charge carriers^12^.

The family of perovskite materials based on Pb^2^⁺ (lead ion) remains essential for ensuring excellent photoelectric properties. However, the byproducts resulting from the breakdown of lead-based perovskites pose environmental hazards to humans and various other species, thereby limiting the mass production of this technology^13^. Therefore, lead-free perovskite light absorbers with reduced toxicity have garnered significant attention recently. Tin and lead are belonging to the same group 14 of the periodic table, allowing for tin to be considered as a promising eco-friendly alternative. Tin halide-based perovskites exhibit advantageous properties such as low exciton binding energy, and narrow band gap with a high carrier mobility^14^. However, the primary challenge faced by tin-based perovskite solar cells (PSCs) lies in their poor air stability due to the susceptibility of Sn^2^⁺ ions to oxidize into Sn^4^⁺ ions. In recent years, various experimental strategies have been employed to prevent this oxidation during both device fabrication and operation^15^. As a result, maximum experimental photoelectric conversion efficiency (PCE) of over 13%, and 7% were obtained for ${\text{FASnI}}_{3}$ and ${\text{MASnI}}_{3}$ based PSCs, respectively. 

Compared to ${\text{MASnI}}_{3}$, the exceptional performance of ${\text{FASnI}}_{3}$ as photoactive absorber in solar cell applications stems from their inherent optical and electrical properties, including a high absorption coefficient, facilitating efficient sunlight harvesting, a long carrier diffusion length for effective charge transport, a small exciton binding energy enabling efficient charge separation, ambipolar charge carrier transport ensuring device stability, and a high tolerance to defects for long-term performance. These originate from the strong interaction of organic cations (FA⁺) with the inorganic framework of ${\text{FASnI}}_{3}$, affecting the band length and band angle distribution, and causing structural changes in ${\text{FASnI}}_{3}$^16^.

Knowing that the theoretical maximum efficiency of perovskite solar cells is approximately 25–27% for a single planner structure, which has not met experimentally yet^17^. Therefore, numerical simulations done using SCAPS-1D can provide an effective cost approach to explore the optimized structure of PSCs in a brief time. Over the years, researchers investigated the photovoltaic characteristics of various materials serving as an electron and hole transporting layer (ETL and HTL) in either normal or inverted structure of FASnI_3_ PSCs. Studied ETLs include TiO_2_, SnO_2_, WS_2_, ZnO, CdS and others^18^. By utilizing mesoporous TiO_2_, several improvements are achieved, such as reduced leakage current, increased recombination lifetime, and hence enhanced Vos and FF^19^.

Regarding HTLs, PEDOT:PSS, Spiro-OMeTAD, PCBM, ${\text{Cu}}_{2}\text{O}$, ${\text{MoO}}_{3}$, Si, and others are studied. Recently, Ritu et al. reported the simulated ${\text{SnO}}_{2}/{\text{FASnI}}_{3}/\text{PCBM}$ PSCs and found a 24.54% PCE (${\text{V}}_{\text{oc}}$ of 0.97 V, ${J}_{\text{sc}}$ of 29.88  mA cm^−2^ and FF of 84.39%)^20^. Almufarij et al. reported higher PCE of 30.45% by inclusion double layers of HTMs of MoO_3_/Si. The double layer enhance the recombination losses in FASnI_3_ achieving 1.143 V, 30.79 mA cm^−2^ and 86.56%, for ${\text{V}}_{\text{oc}}$, ${J}_{\text{sc}}$, and FF , respectively^18^.

In this study, we report the optimization of FASnI_3_ perovskite solar cells with four different hole transporting materials using the SCAPS-1D software^12,18^. These are Spiro-OMeTAD, Cu_2_O, CuI, and CuSCN. The effects of absorber layer thickness, defect density states, work function of the back metal contact, and light properties at surfaces and interfaces, particularly the light reflection/transmission ratios at the back/front contact, and the capture cross-sections for charge carriers are investigated. The photovoltaic outputs, including JV, QE, and energy band structure, are analyzed.

## Material and methods

### SCAPS-1D software

The analysis of optoelectrical modelling and photovoltaic characteristics involves utilizing SCAPS software. This reliable program has recently gained popularity within the photovoltaic community for modelling perovskite devices, and the obtained output aligns well with experimental data^21^. An inherent electric field normal to the interfaces is induced by the alignment of energy levels at the interfaces caused by the asymmetric designs of devices, which result in a work function offset between the anode and cathode. This arrangement helps charge carriers drift, making collecting them at electrode contacts easier^22,23^.

There are supposed to be neutral defects at the mid-band gap level in the model, with a Gaussian distribution and a characteristic energy level of 0.10 eV. Cell performance is assessed by utilizing the Shockley–Read–Hall (SRH) recombination model to thoroughly analyze the effects of defect density (${N}_{T}$) and capture cross sections (${\sigma }_{n,p}$) for charge carriers at the interface and bulk levels of the absorber layer. The following describes the SRH recombination model^24^.

1 $${R}^{SRH}=\frac{{\upsilon }_{th}{\sigma }_{n,p}{N}_{t}[np-{n}_{i}^{2}]}{n+p+2{n}_{i}\text{cosh}\left(\frac{{E}_{t}-{E}_{i}}{kT}\right)}$$

The ${\sigma }_{n,p}$ is contingent on their lifetime (${\tau }_{n,p}$) before undergoing recombination to form an exciton, as expressed below:

2 $${\sigma }_{n,p}=\frac{1}{{\tau }_{n,p}{\upsilon }_{th}{N}_{t}}$$

3 $${\tau }_{n,p}= \frac{1}{{\sigma }_{n,p}{\upsilon }_{th}{N}_{t}}$$

The symbols *p* and *n* represent the densities of electrons and holes under non-equilibrium conditions. The intrinsic density (n_i_), intrinsic energy level (E_i_), energy level of trap defects (E_t_), and thermal velocity of electrons and holes (*v*_th_) are the characteristics that are used to evaluate the performance of solar cells. These characteristics include fill factor (FF), power conversion efficiency (PCE), short-circuit current density (J_sc_), open-circuit voltage (V_oc_), quantum efficiency (QE), and current–voltage (J–V)^25^.

4 $$i={i}_{ph}-{i}_{o}\left[\text{exp}\left(\frac{qV}{\beta kT}\right)-1\right]$$

where *i*_*o*_ represents the dark saturation current.

5 $${i}_{o}=q\left(\frac{{D}_{n}{n}_{i}^{2}}{{L}_{n}{N}_{A}}+\frac{{D}_{P}{n}_{i}^{2}}{{L}_{p}{N}_{D}}\right)$$

The following formula can be used to estimate the V_oc_^26^:

6 $${V}_{OC}= \frac{akt}{q}\text{ln}\left(\frac{{i}_{ph}}{{i}_{o}}+1\right)$$

where *(kt/q)* is the thermal voltage and *a* is factor. The non-radiative and radiative recombination occurring in the primary exciton production zone determines the EQE, which is represented as:

7 $${EQE}_{EL}=\frac{{J}_{o,rad-bi}}{{J}_{o,rad-bi}+{J}_{o,nr-bi}+{J}_{o,nr-trap}}$$

where ${J}_{o,rad-bi}$ indicates the background current density arising from the radiative part of molecular recombination, ${J}_{o,nr-bi}$ indicates the background current density originating from the nonradiative part of molecular recombination, and ${J}_{o,nr-trap}$ indicates the background current density linked to non-radiative trapassisted recombination.

### Structure of FASnI_3_-based perovskite solar cells

As highlighted in the introduction ABX_3_ general formula, utilizing inorganic–organic absorber layers in perovskite devices presents a significant challenge related to their long-term operational stability. This concern extends to the material phase stability of the device during its preparation, growth, and synthesis with the risk of limiting large-scale production. The low phase stability observed in organic–inorganic perovskite crystals is typically associated with the halide component^27,28^. This rationale supports the selection of formamidinium (FA) or ethyl-ammonium organic cations similar to the conventional property of common methylammonium (MA)^29,30^. Introducing FA into MA-based perovskite has resulted in notable thermal and environmental stability improvements and enhanced device performance. In addressing toxicity concerns, the divalent tin cation (Sn^+2^) has garnered attention as a practical substitute for the unstable and hazardous lead (Pb). However, one drawback of Sn-based devices is their susceptibility to rapid oxidation, wherein Sn^+2^ readily oxidizes to Sn^+4^ upon exposure to air, resulting in swift degradation of cell properties^31,32^. Compared to Pb^+2^, Sn^+2^ has a smaller atomic diameter, improved conductivity, and the capability to form stable perovskite structures. Shao et al. reported high stability with maximum performance of Sn-based PSC and the highest reproducibility achieved^33,34^.

A high-quality FASnI_3_ layer with uniform surface morphology and excellent crystallinity is possible through various deposition techniques, including chemical solution deposition, co-evaporation deposition and vapor-chemical solution deposition^14,35^. In this work, however, the structure of FASnI_3_—based PSCs, modelled using SCAPS, is FTO/TiO_2_/FASnI_3_/HTL/Ag, where TiO_2_ is the electron transport layer (ETL), FASnI_3_ is the absorber material, HTL is the hole transporting layer, and Ag is the anode.

### Device modelling

**Figure 1:**
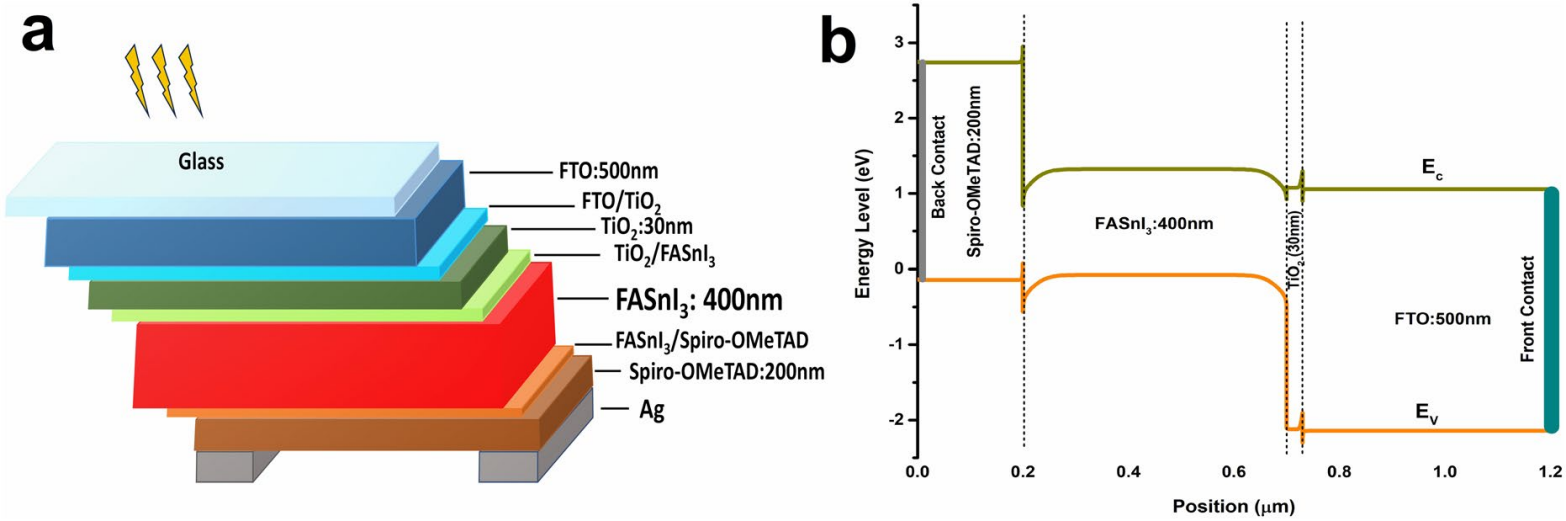
(**a**) schematic diagram and (**b**) energy band diagram of FASnI_3_ based device structure.

First, four different hole transporting materials (HTM) were employed due to their similar electronic and optical properties. These included Spiro-OMeTAD, CuI, Cu_2_O, and CuSCN. However, the initial proposed device was based on Spiro-OMeTAD. It had the following configuration of FTO/TiO_2_/FASnI_3_/Spiro-OMeTAD/Ag, as shown in Fig. 1a, while Fig. 1b showed its energy band diagram. Input parameters of the initial structure were taken from the literature and shown in Table 1. Other HTMs had also similar device configurations with input parameters listed in Table 2.

**Table 1:** input parameters of the initial proposed device.

Properties	Sprio-OMeTAD	FASnI_3_	TiO_2_	FTO
Thickness (µm)	0.20	0.40	0.030	0.50
E_g_ (eV)	2.88	1.4	3.2	3.2
χ (eV)	2.05	4.17	4.0	4.4
ε_r_ (eV)	3.0	8.2	100	9.0
N_V_ (1/cm^3^)	2.5 × 10^20^	1.0 × 10^18^	2.0 × 10^20^	1.8 × 10^19^
N_C_ (1/cm^3^)	2.5 × 10^20^	1.0 × 10^18^	1.0 × 10^21^	2.2 × 10^18^
µ_e_ (cm^2^/Vs)	2.1 × 10^−3^	22	6.0 × 10^−3^	20
µ_p_ (cm^2^/Vs)	2.1 × 10^−3^	22	6.0 × 10^−3^	10
N_D_ (1/cm^3^)	0	1.0 × 10^18^	5.06 × 10^19^	1.0 × 10^19^
N_A_ (1/cm^3^)	1.0 × 10^18^	0	0	0
N_T_ (1/cm^3^)	10^15^	10^15^	10^15^	10^14^
Reference	^37^	^38^	^37^	^37^

**Table 2:** input parameters of the different HTLs.

Properties	CuSCN	CuI	Cu_2_O
Thickness (µm)	0.20	0.20	0.20
E_g_ (eV)	3.4	2.98	2.17
χ (eV)	1.7	2.1	3
ε_r_ (eV)	10.0	6.5	7.5
N_V_ (1/cm^3^)	1.8 × 10^18^	1.0 × 10^19^	1.1 × 10^19^
N_C_ (1/cm^3^)	2.2 × 10^19^	2.8 × 10^19^	2.0 × 10^18^
µ_e_ (cm^2^/Vs)	100	100	200
µ_p_ (cm^2^/Vs)	25	43.9	80
N_D_ (1/cm^3^)	0	0	0
N_A_ (1/cm^3^)	2.0 × 10^19^	2.0 × 10^19^	2.0 × 10^19^
N_T_ (1/cm^3^)	10^15^	10^15^	10^14^
References	^37^	^37^	^37^

Second, other parameters like thickness of the active layer, the work function of metal back contact were varied and investigated. Third, the ETL/FASnI_3_ and FASnI_3_/HTL interfaces were simulated via varying capture cross-sections for electrons and holes, structural and interface defect density states, which were assumed to account for both structural and interface recombination ratio. Neglecting these interfaces could result in erratic outcomes due to high discontinuity between the absorber layer and ETL/HTL. Optimization calculations were then performed, simulating the effects of front and back contacts on light transmission and reflection. Finally, the diagram of energy band levels was drawn for the optimized FASnI_3_ PSCs.

The effects of all previous parameters were evaluated by analyzing the evolution of J–V and QE properties. It should be noted that every simulation was run with 1000 Wm^–2^ of external light at 1.5 G AM and 300 K operational temperature. The electron and hole thermal velocities were both set at 10^7^ cm/s. With a Gaussian distribution, defects were regarded as neutral^36^.

## Results and discussion

### Effect of different hole transporting materials

**Figure 2:**
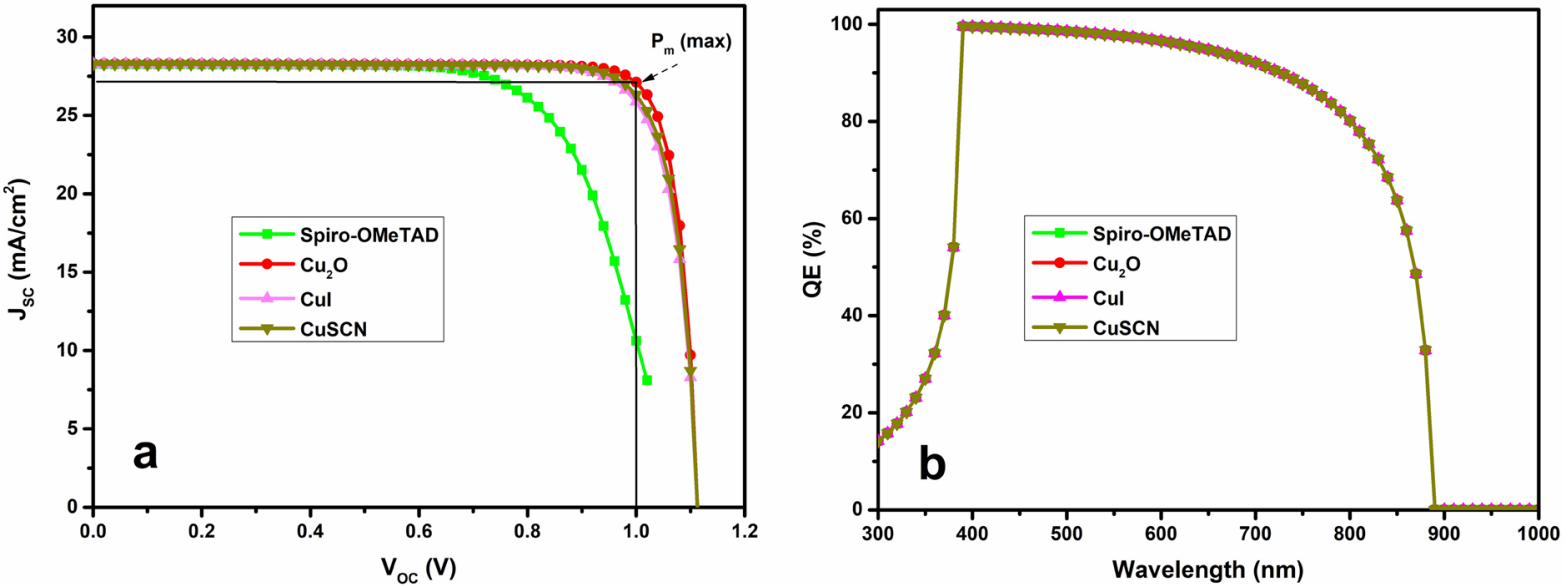
The J–V and QE properties of the four HTLs.

In solar cell structure, the hole transporting material (HTM) serves two primary functions: (1) giving the photogenerated holes in the active layer a reachable energy level to enable quick transport throughout the circuit, avoid recombination, and (2) preventing electrons that are rejected by an energy barrier that is high enough. The material selected for the HTL has a substantial impact on cell performance since holes determine the p-type conductivity of perovskite. When selecting the appropriate HTM, key features consist of the valence band states, charge carrier density, dielectric constant, and energy gap. Numerous studies have investigated the impact of HTL on photovoltaic outputs. This section uses a relative analysis to evaluate the influence of incorporating various hole transport materials on current–voltage and external quantum characteristics. In the initial cell employs Spiro-OMeTAD, while the other three structures use CuSCN, CuI and Cu_2_O, with key property values summarized in Table 2. Figure 2 shows that the structure incorporating Cu_2_O as the HTM demonstrates superior performance, as depicted by its current–voltage curve, which has the greatest mean power. Furthermore, Table 3 indicates that the optimal performances, with PCE of 27.13% and FF of 86.08%, are achieved. The hole mobility, with a magnitude of 80 cm^2^/Vs, is approximately 2000 times larger than that of SPO, explaining the increased hole flow. Additionally, its dielectric constant (7.50 eV) is greater than two times of SPO, influencing the strength between charge carriers and contributing to the high hole mobility. Since the HTL has a smaller energy gap (2.17 eV) than SPO (2.88 eV) and the maximum range of incident light reaching it, has less of an impact on QE. It only marginally contributes to the formation of carriers^39,40^. Consequently, the QE curves of all cells are all overlapping.

**Table 3:** The comparative performance of devices with different HTLs.

Structure of PSCs	V_OC_ (V)	J_SC_ (mA/cm^2^)	FF (%)	PCE (%)
Ag/Spiro-OMeTAD/FASnI_3_/TiO_2_/FTO	1.08	28.25	68.4	20.95
Ag/Cu_2_O/FASnI_3_/TiO_2_/FTO	1.11	28.30	86.08	27.13
Ag/CuI/FASnI_3_/TiO_2_/FTO	1.11	28.28	82.93	26.12
Ag/CuSCN/FASnI_3_/TiO_2_/FTO	1.11	28.28	84.02	26.46

### Effect of absorber layer thickness

**Figure 3:**
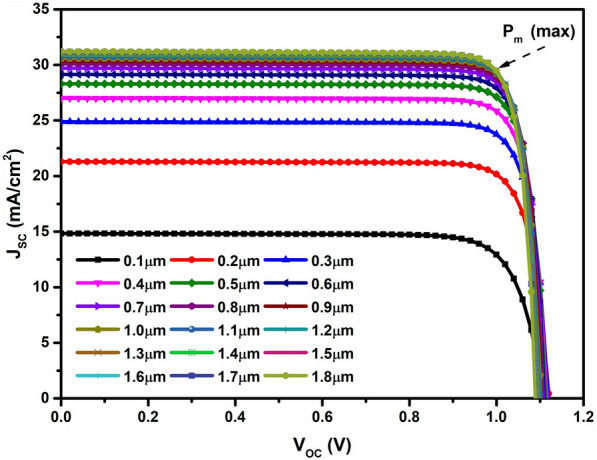
The J–V characteristics of the PSCs with changing the perovskite layer thickness.

**Figure 4:**
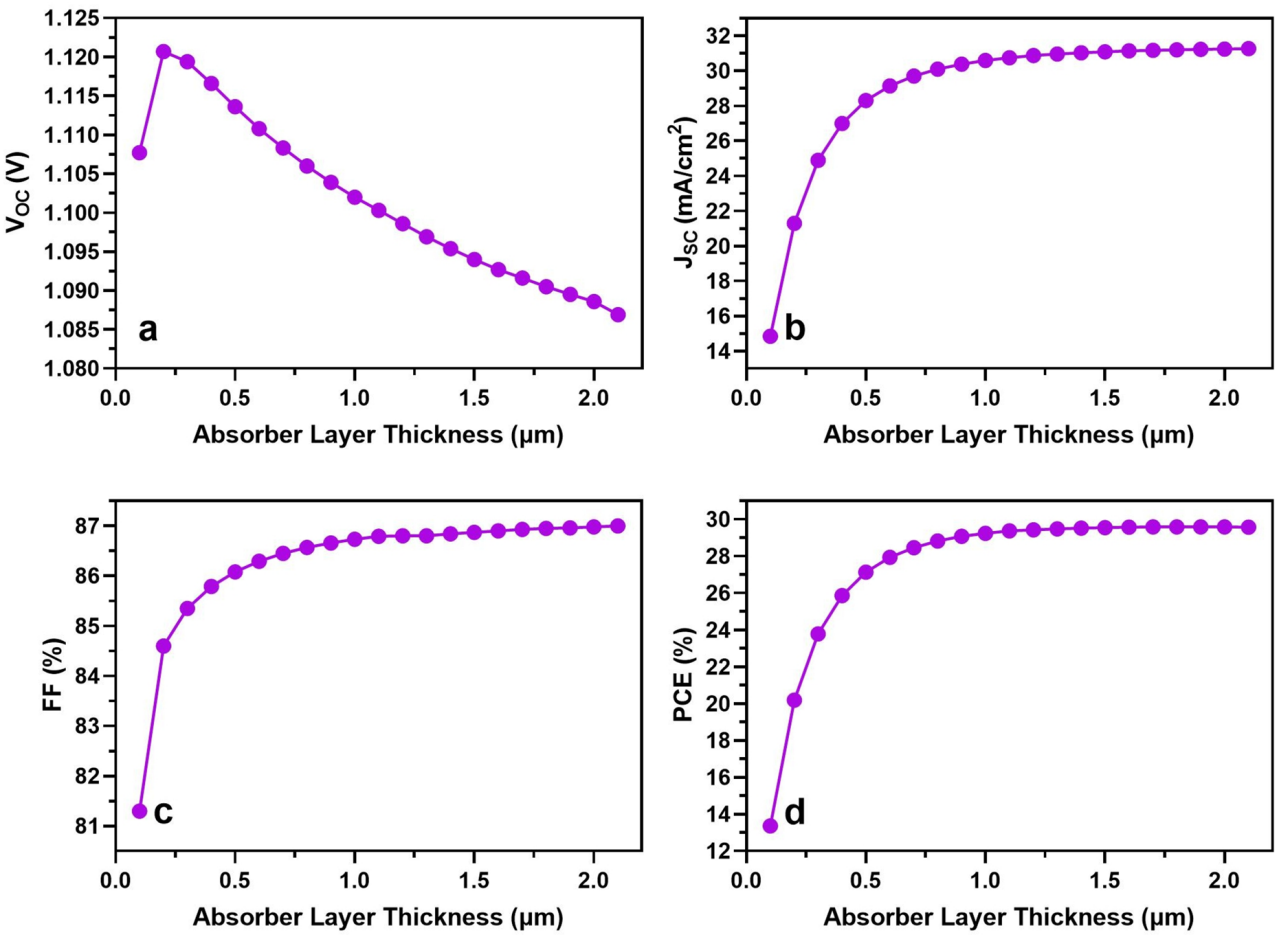
Impact of performance layer thickness on device efficiency.

Perovskite cells are categorized as thin film devices, typically featuring an active layer thickness in the moderate range with maximum light absorption. They are recognized for achieving high yields even with a thin layer. As illustrated in Fig. 3, this is evident in the progression of J–V curves. Figure 4 shows the performance of PSCs with changing the perovskite layer thickness from 0.1 to 1.8 µm. Particularly, the most efficient devices are observed within the active layer thickness range of 1.5–1.7 µm, based on J–V curves exhibiting the maximum mean power (P_m_ = I_m_ × V_m_). We were able to choose 1.6 µm in the further simulations.

The effectiveness of these devices within this thickness range is attributed to the extended duration of light within the device material when the perovskite layer is thicker than the n-type layer. This results in a longer optical path length for the light within the absorber layer, increasing the probability of photon absorption and the generation of more charge carriers^41^. Consequently, this leads to an enhancement in cell conversion efficiency. On the contrary, when these values are exceeded by the active layer’s depth, the J–V characteristics indicate almost similar efficient devices^40,42^. The advantage of moderate thickness includes a shorter deposition time, lowering the overall process cost.

### Effect of defect density states

It is possible to categorize defects in perovskite solar cells as either impurities or interruptions in the pure crystal structure. Since defects emerge during the deposition, or growth routes, material preparation, their inclusion in the construction is inevitable. For more accurate results, numerical modelling of a device involves considering its defect density in the interface or volume. This study investigates the impact of defects states density on PSC performance by changing N_T_ from 10^14^ to 10^18^ cm^−3^, representing a range from theoretical device to a heavily tainted material. We have optimized the defect density state of the absorber layer 10^14^ cm^−3^^43^.

**Figure 5:**
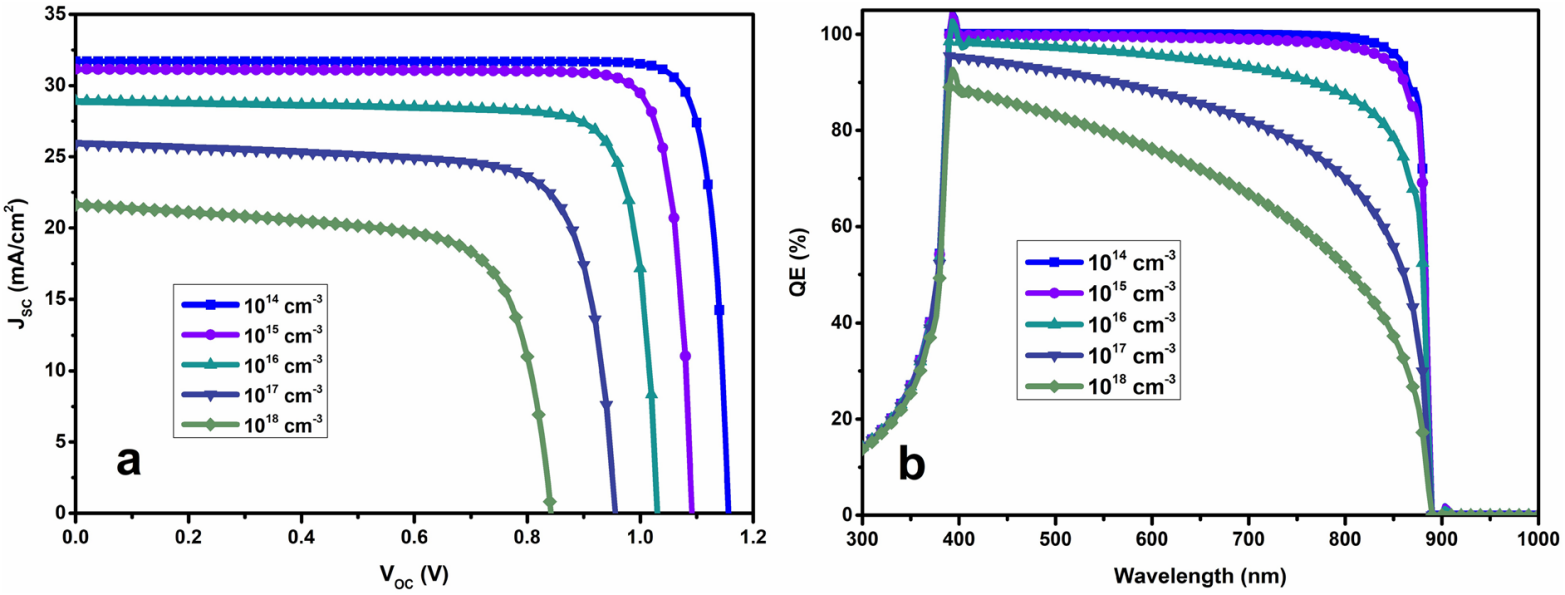
Effect defect density state of perovskite layer in PSCs.

As depicted in Fig. 5, the external quantum efficiency of the cell decreases from nearly 99% to 75% as N_T_ increases. This means that just 25% of the light is engaged in the cell structure. Increased bulk non-radiative charge carrier recombination occurs in the absorber layer of cell because of deeper trap sites for charge carriers created by an increase in N_T_ at the bulk and surface defect levels, or grain boundaries. The defects located inside the device structure and materials with defect density up to 10^15^ cm^−3^ can function efficiently. Identifying the tolerance range for impurities in the perovskite layer is crucial for effective functioning, it is pertinent to research including experiments^44^. Moreover, this study aligns with the findings from the literature, indicating that achieving devices with low N_T_ values which is less than 10^14^ cm^−3^ in experiments, is challenging with current production methods. It has been reported N_T_ of 10^14^ cm^−3^ as an optimum value.

### Effect of light reflection/transmission at the back/front contact

Overly optimistic simulation of the cell would arise from assuming full transmission of the incoming light flux at the front contact or no light reflection at the interior layers, including the back contact. It is not possible to totally transfer all light into the absorber layer; part of it will always be lost because of absorption in the layers that come before it, including TiO_2_ and FTO. The light transmittance can only be accurately modelled at the FTO, serves as the front contact, using SCAPS.

**Figure 6:**
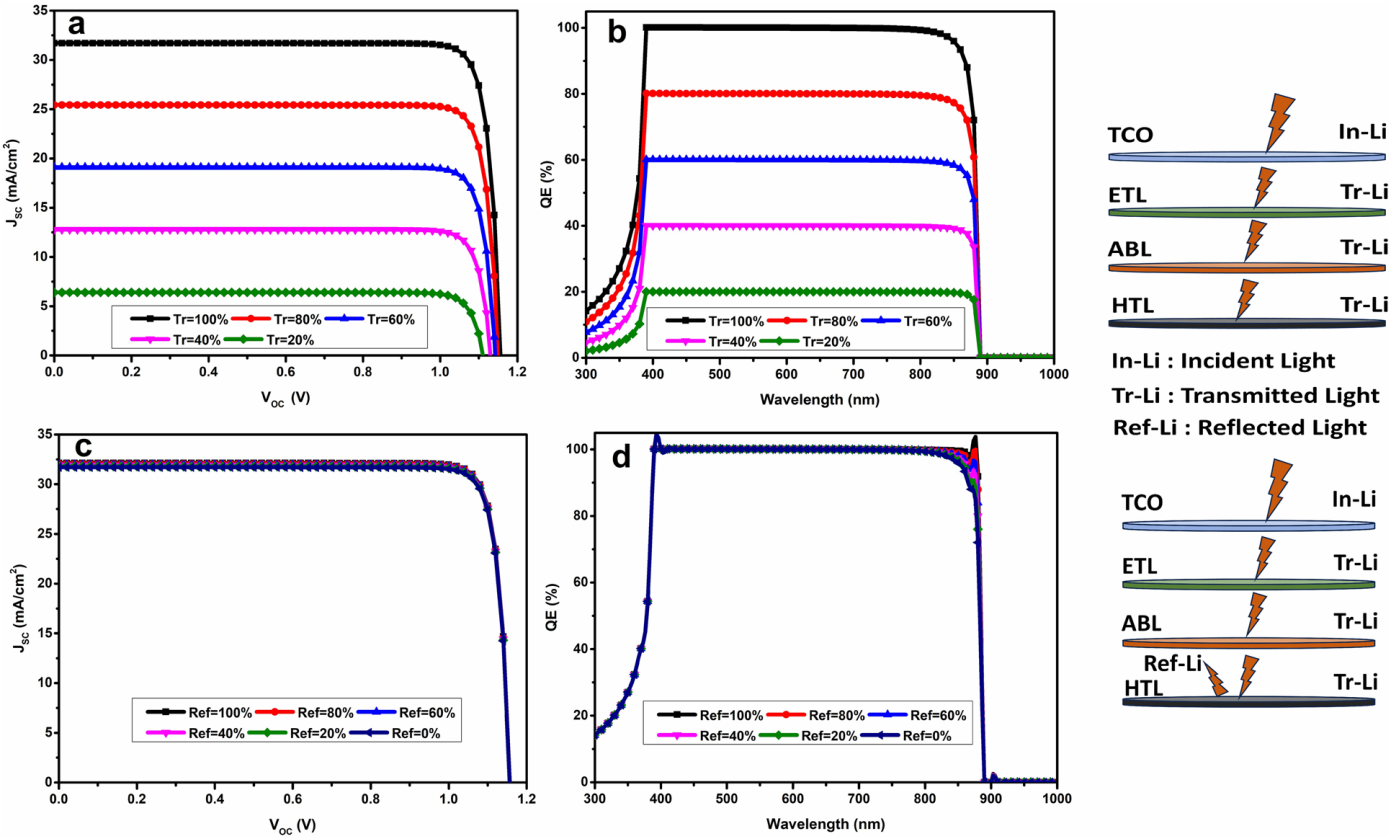
The influence of the light reflection/transmission ratio at back/front contact on the performance of FASnI_3_-based PSCs.

Figure 6a,b show the trends of the J–V and QE properties of the device as the percentage of light transmittance through the front contact varies from 20 to 100%. This feature has a negligible impact on V_oc_, remaining within the range [1.1; 1.15]. However, J_sc_ is significantly affected, dropping from 31.3 to 6.2 mA/cm^2^ as the transmission drops from 100 to 20%. Similarly, EQ decreases from 99 to 20%. The production of large charge carriers and, subsequently, the current are affected when the transmission rate drops because fewer photons reach the transmission layer.

Recombination is necessary for the few photogenerated carriers to reach the splitting interface because of the active layer’s thickness of 1.6 µm. Reducing the thickness of the absorber layer within the ideal range measured between 1.5 and 1.7 µm is one possible scenario to make up for the limited light transmission via the front layer and contact. Fortunately, a large enough region of the visible spectrum can pass through the absorber layer since the gap between the front window and contact layers is designed to be suitably high (3.2 eV). A device made from non-transparent FTO and ETL materials would exhibit inferior performance.

Furthermore, each inner layer’s surface reflects tiny light beams, which begs the question of whether the performance of the cell is directly impacted by this reflected light. Only the reflection % at the back contact is considered by SCAPS. Significant incoming light is trapped by FASnI_3_ with a band gap of 1.4 eV. The active layer reabsorbs light that is reflected at the back surface when it escapes and heads towards it. This increases the active layer’s absorption capacity and produces more charge carriers, which raise the photogenerated current and, in turn, the cell’s overall current. As seen in Fig. 6c,d, which depict the evolution of J–V and QE features, this augmentation is especially noticeable in the present. When the reflection percentage increases from 20 to 100%, V_oc_ stays constant while the current significantly changes from 31 to 32 mA/cm^2^. Better QE is seen in the 700–1000 nm spectral range, which primarily corresponds to the portion of light that was not absorbed during the active layers first trip.

This conclusion is relevant and consistent with earlier studies considering how important it is to build solar cell with reflecting surfaces at the back metal contact^11^. These findings highlight how crucial it is to use materials with maximum transmittance rates in order to maximize solar spectrum transmission.

### Effect of metal work function

In perovskite solar cell devices, at least one electrode requires a low work function (WF) to inject or collect charge carriers into the conduction band. To effectively capture holes separated at the FASnI_3_/ETL interface, back metal contacts should have a greater WF. A higher WF of the back contact enhances hole collection efficiency, allowing easy migration from the hole transport layer to the maximum energy level. Nonetheless, electrons must be prevented from passing through the WF of the back metal contact. It was calculated that 4.8 eV would be the ideal WF for metal back contact.

**Figure 7:**
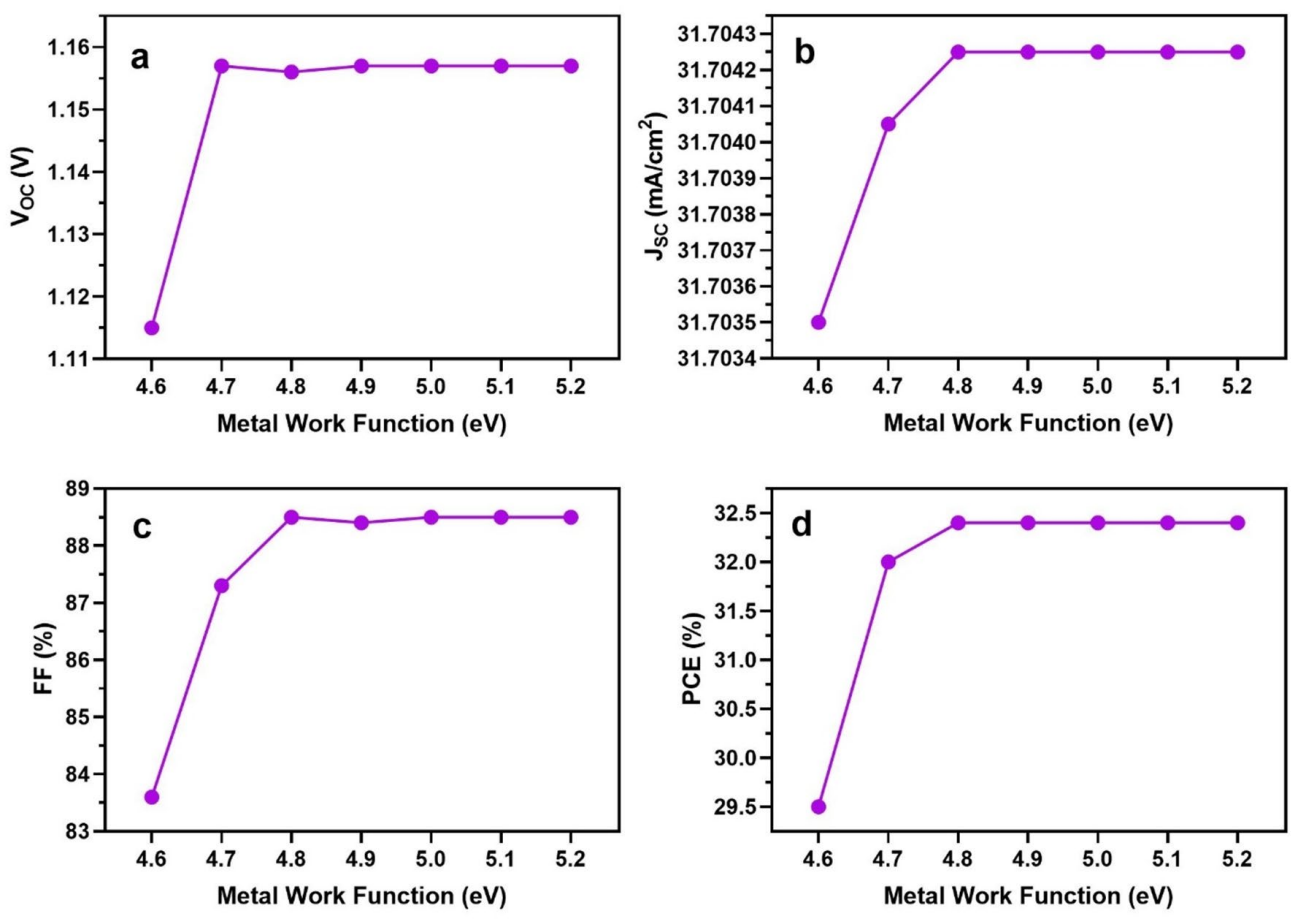
Output IV parameters of FASnI_3_-based PSCs with the work functions of the metal back contact.

As shown in Fig. 7, the maximum performance of the device was obtained with WF values greater than the value of 4.8 eV. Simulations consistently produced steady results above this threshold value. This is emphasized in Fig. 7, where V_oc_, J_sc_, FF and PCE of all remain nearly constant once the WF of the metal contact surpasses 4.8 eV. These results attribute the increased built-in potential to the better V_oc_ and overall efficiency of their PSC device.

### Effect of charge carriers’ capture cross-sections

In this section, we conducted modelling assuming equal capture cross-sections for electrons and holes (i.e., ${\sigma }_{n, p}={\sigma }_{n}={\sigma }_{p}$). The numerical analysis focused on different volumetric capture cross-sections at FTO/TiO_2_, TiO_2_/FASnI_3_ and FASnI_3_/Cu_2_O interfaces. The effective cross-section of trap levels between the valence and conduction bands that may capture photogenerated carriers at a specific thermal velocity (V_th_) is measured by the capture cross-section.

**Figure 8:**
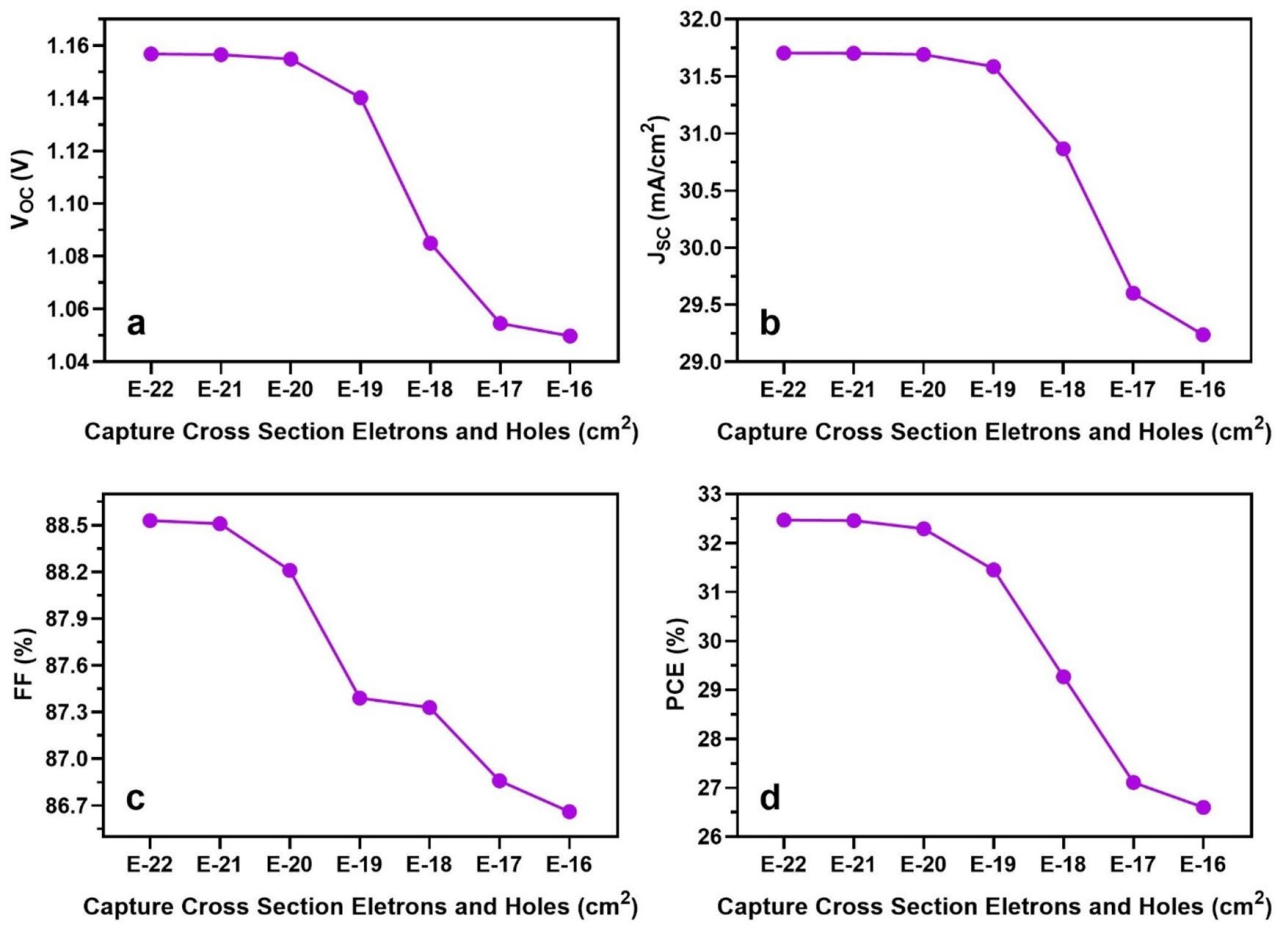
Effect of varying capture cross-sections on the absorber layer FASnI_3_ and interfaces FTO/TiO_2_, TiO_2_/FASnI_3_ and FASnI_3_/Cu_2_O on output parameters.

An approximate defect density of 1 × 10^15^ cm^−3^ was taken into account in the volume and at the interfaces, in line with earlier calculations. The numerical simulations were carried out by changing the ${\sigma }_{n, p}$ from 1 × 10^16^ to 1 × 10^22^ cm^2^^45^. As shown in Fig. 8, all photovoltaic output IV parameters are strongly affected by the variation of ${\sigma }_{n, p}$. A smaller ${\sigma }_{n, p}$ indicates a more effective structure. According to about mentioned equation of ${\sigma }_{n, p}$, as the ${\sigma }_{n, p}$ decreases. Additionally, there is a considerable decrease in Shockley–Read–Hall recombination current, which improves the open-circuit voltage, lowers cell losses, and improves device performance.

Maintaining a constant defect density N_T_ and thermal conditions of simulation (V_th_ = C^te^), a reduction in ${\sigma }_{n, p}$ leads to a longer lifespan for charge carriers, enhancing their contribution to the photo generation of current. Varying ${\sigma }_{n, p}$ from 1 × 10^16^ to 1 × 10^22^ cm^2^ increases V_oc_ from 1.05 to 1.16 V, while the J_sc_ value increases from 29.23 to 31.70 mA/cm^2^. This leads to exceptional results with a PCE of 32.47% and FF of 88.53%.

From literature the authors have achieved the maximum performance of 26.33% with their numerical modelling with 1.0 μm thickness of the device with the ${\sigma }_{n, p}$ = 1 × 10^19^ cm^2^^46^. Effective passivation at the interface layer can result in small ${\sigma }_{n, p}$, which attenuate bulk defects and minimize grain size variations at thresholds.

### Energy band diagram of optimized structure

**Figure 9:**
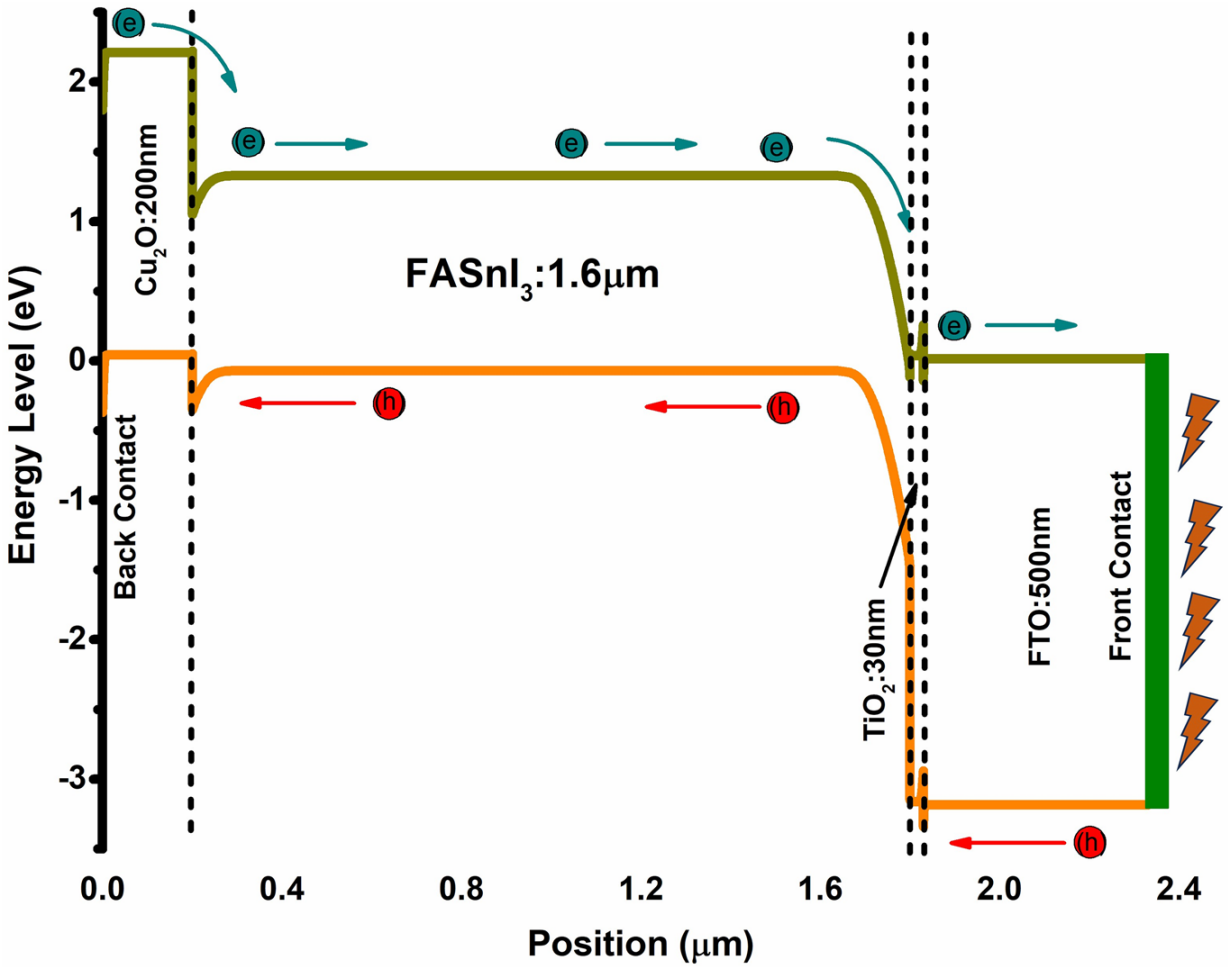
Energy band diagram illustrating the Ag/Cu_2_O/FASnI_3_/TiO_2_/FTO structure with the flow of charge carriers.

The transfer of photogenerated charge carriers across the device is demonstrated by charge carrier transport, which is depicted in Fig. 9 of the optimized devices energy level diagram. Electrons can migrate easily from higher to lower energy levels, such as Cu_2_O (E_C_ ~ 2.65 eV) to FASnI_3_ (E_C_ ~ [0.5: 1.4 eV]), TiO_2_ (E_C_ ~ 0.50 eV), and finally FTO (E_C_ ~ 0.20 eV). Similarly, the valence level arrangement is responsible for successful hole transport: the layer below the most valence-energy-rich layer for practical participation in charge carrier photogeneration. Holes can move freely from FTO (E_V_ ~ − 3.20 eV) to TiO_2_ (E_V_ ~ − 3.10 eV), then to FASnI_3_ (E_V_ ~ [− 1.3: − 0.15 eV]), and finally to Cu_2_O (E_V_ ~ 0 eV). The formation of a spike between the TiO_2_ and FTO impedes the movement of photogenerated electrons toward the front electrode^29,31^.

## Conclusion

The FASnI_3_ -based perovskite solar cells with an initial structure of maximum performance of 20.95% and FF of 66.40% have been theoretically achieved using SCAPS-1D software. To address industry challenges such as stability, cost efficiency, and more moderate thickness of the device, optimization calculations were conducted by varying material properties in the functional layers, focusing on assessing the impact on output characteristics through J–V and QE analyses. The results demonstrated substantial improvements, and an investigation into the effects of capture cross-sections of the holes and electrons on PV properties led to achieving a PCE of 32.47% and an FF of 88.53% using cell with ${\sigma }_{n, p}$ = 1 × 10^22^ cm^2^. The optimization approach shows promise, identifying Cu_2_O as a prospective material for stable and highly efficient PSCs, reducing capture cross-sections of electrons and holes through efficient passivation and minimizing defect density. The proposed architecture for future PSCs is Ag/Cu_2_O/FASnI_3_/TiO_2_/FTO.

## Data Availability

The datasets generated and analyzed during the current study are available from the corresponding author upon reasonable request.
